# Subcellular Proteomics: Application to Elucidation of Flooding-Response Mechanisms in Soybean

**DOI:** 10.3390/proteomes6010013

**Published:** 2018-02-27

**Authors:** Setsuko Komatsu, Akiko Hashiguchi

**Affiliations:** 1 Faculty of Life and Environmental Sciences, University of Tsukuba, Tsukuba 305-8572, Japan; 2 Faculty of Medicine, University of Tsukuba, Tsukuba 305-8577, Japan; hashiguchi.akiko.ge@un.tsukuba.ac.jp

**Keywords:** subcellular proteomics, flooding-response mechanism, soybean

## Abstract

Soybean, which is rich in protein and oil, is cultivated in several climatic zones; however, its growth is markedly decreased by flooding. Proteomics is a useful tool for understanding the flooding-response mechanism in soybean. Subcellular proteomics has the potential to elucidate localized cellular responses and investigate communications among subcellular components during plant growth and during stress. Under flooding, proteins related to signaling, stress and the antioxidative system are increased in the plasma membrane; scavenging enzymes for reactive-oxygen species are suppressed in the cell wall; protein translation is suppressed through inhibition of proteins related to preribosome biogenesis and mRNA processing in the nucleus; levels of proteins involved in the electron transport chain are reduced in the mitochondrion; and levels of proteins related to protein folding are decreased in the endoplasmic reticulum. This review discusses the advantages of a gel-free/label-free proteomic technique and methods of plant subcellular purification. It also summarizes cellular events in soybean under flooding and discusses future prospects for generation of flooding-tolerant soybean.

## 1. Introduction

Soybean is an important legume crop due to its high content of protein and vegetable oil. The production and consumption of soybean are gradually increasing worldwide. Soybean is susceptible to flooding stress [1], which is a major problem that affects seed germination, plant growth and seed yield [2]. Early exposure of soybean plants to flooding stress causes severe damage due to rapid imbibition of water by the cotyledon and destruction of the root system [3]. The shortage of oxygen under flooding stress results in a shift from aerobic to anaerobic respiration and leads to a shift to alternative pathways of energy generation. The low diffusion rate of oxygen under flooding stress is a limiting factor for plant survival and most plants die under limited oxygen supply [4]. In soybean, flooding stress mainly suppresses plant growth not only by impairing root elongation but also by reducing hypocotyl pigmentation [5], which leads to a low intracellular oxygen level and synthesis of proteins related to anaerobic metabolic pathways [6]. To understand the flooding-response mechanism in soybean, proteomic techniques are useful because flooding leads to comprehensive stress.

In soybean seedlings under flooding stress, Mutava et al. [7] reported that fibrillin proteins have a potential role. Furthermore, proteomic techniques revealed changes in proteins involved in hormonal signaling, transcriptional control, glucose degradation and sucrose accumulation, glycolysis, alcohol fermentation, the gamma-aminobutyric acid shunt, mitochondrial impairment, ubiquitin- and proteasome-mediated proteolysis, cell wall loosening and active oxygen scavenging [8]. Additionally, to investigate how soybeans respond to flooding with post-translational modifications, gel-based and gel-free proteomic techniques have been used [9]. Flooding stress induced changes in post-translational modifications such as glycosylation [10,11], ubiquitination [12] and phosphorylation [13,14,15,16], which are common signaling events occurring in response to stress.

To obtain comprehensive knowledge, Chen et al. [17] used transcriptomic technique and reported that the changes in expression of genes involved in regulating the flux of cell wall precursors and starch/sugar content can serve as an adaptive mechanism for soybean survival under flooding stress. Additionally, for obtaining the cellular reactions involved in stress-response mechanisms, the role of cellular organelles should be considered in soybean under flooding [18,19]. Soybean proteins affected by flooding stress were identified in subcellular organelles such as the nucleus [16,20,21,22,23], mitochondria [10,24,25], endoplasmic reticulum [11,26], cell wall [27] and plasma membrane [28]. In this review, advantages of a gel-free/label-free proteomic technique are discussed and methods of plant subcellular purification are described. Here, subcellular organelles that are central in biological processes against flooding in soybean were chosen to describe the application of subcellular protein purification in plants to allow further practice in this area. Furthermore, the cellular events in soybean under flooding are summarized and future prospects for generation of flooding-tolerant soybean are discussed.

## 2. Strengths and Problems of Subcellular Proteomic Techniques

### 2.1. Gel-Free and Gel-Based Techniques

Protein quantification based on the two-dimensional gel electrophoresis method established by O’Farrell [29] is the most common technique for proteomic analysis. This technique separates proteins in two steps: the first dimension is isoelectric focusing, which separates proteins according to their isoelectric points; during the second dimension the proteins are separated according to molecular weight by SDS-polyacrylamide gel electrophoresis. Further sensitivity is achieved by covalent labeling of proteins with fluorescent Cy-dyes before separation. This method, called two-dimensional difference gel electrophoresis, improves the quality and number of protein spots and provides more reliable gel matching [30]. However, these gel-based techniques are fairly insensitive to proteins that exist in small amounts and are limited in proteome resolution [31]. Another disadvantage of these methods is the difficulty in detection of different types of post-translational modifications of a single protein that result in crosstalk among signal pathways. Because capturing subtle changes in the entire proteome is essential for elaborate studies of plant physiology, non-gel-based techniques that comprise in-solution protein digestion have been developed [32]. Solubilization of membranous proteins remains as a limitation in membrane proteomics using gel-free methods due to different optimum condition [33] but data on membrane protein repertoire are accumulating [34]. A gel-free technique has been applied to many plant species including barley [35], soybean [36] and pine [37], further justifying the potential of this method by the identification of a far larger number of proteins. Used as complementary approaches, gel-based and gel-free protein quantification will be useful for analyses of the regulatory mechanisms used by plants. Using gel-based, label-free protein quantification, PI3K-mediated vesicular transport has been detected in *Arabidopsis* [38]. Careful selection of proteomic approaches and cellular events that should be resolved by the approach will be a key for studies on plant stress responses.

### 2.2. Label-Free and Label-Based Techniques in Gel-Free Proteomics

The mass spectrometry (MS)-based quantification strategy supports both relative and absolute protein quantification [39]. Metabolic in vivo labeling techniques such as SILAC (stable isotope labeling with amino acids in cell culture) and ^15^N labeling allow quantification with smaller measurement bias. ICAT (isotope-coded affinity tag), ^18^O labeling, TMT (tandem mass tags) and iTRAQ (isobaric tags for relative and absolute quantification) are chemical in vitro labeling methods that can be applied to static samples like clinical samples [40]. TMT and iTRAQ are now the most widely used labeling techniques because they can be used for differential quantification of various protein post-translational modifications [40]. The iTRAQ-based differential proteomics of nuclear proteins using a tomato mutant line revealed that the defects in SlUBC32, a ubiquitin E2 enzyme and PSMD2, a 26S proteasome regulatory subunit, are the cause of ripening inhibition in tomato [41]. The iTRAQ-based strategy was also used in grass species such as Italian ryegrass and black cottonwood to identify Golgi proteome [42]. However, limitations of label-based techniques often come with the experimental design for sample comparison so that there are a few studies using iTRAQ-based strategy in soybean, especially in the field of stress response research which requires comparison among multiple conditions [43]. Applications of iTRAQ-based technique to subcellular compartments are also limited by the cost of reagents and the complex sample preparation [44]. In contrast, label-free quantitation has no limits regarding the number of samples for analysis [44]. In label-free quantitation using MS/MS, the digested peptides are separated by liquid chromatography (LC), transferred to a first mass spectrometer (MS1) where the chromatograms depicting signal intensities are retrieved to measure abundance of each peptide. The peptide ions are selected for further fragmentation in MS2 to identify the parent ion [44]. Label-free LC-MS/MS allows wide quantification of proteins. Simple sample preparation and shorter time demands for sample preparation of gel-free, label-free quantification allowed accumulation of vast amount of data in soybean proteomics, revealing central responses of soybean against flooding. For subcellular proteomics in soybean, changes in nuclear factors suggested the importance of abscisic acid-related signal transduction [16], which controls initial stage of the plant’s response against flooding stress. Discoveries in other subcellular compartments will be discussed in Section 4. However, the remaining issue in this method is optimization of LC-MS chromatogram alignment for accurate quantification. Many platforms are available that use MS/MS scan times or base peak information to align chromatograms [45]. The advantage of gel-free, label-free proteomics resides in its simplicity in sample preparation and the easiness for data production. Large-scale data analysis of accumulated data on protein abundance [46] will lead to elucidation of biological processes that were neglected in small-scale experiments.

## 3. Purification Techniques for Subcellular Proteins from Plants

### 3.1. Nuclei

Subcellular fractionation is important for the detection of organelle component proteins as well as for analysis of low-abundant proteins or isolation of enzymatic complexes. The nucleus is essential for gene expression and regulation [47]. Purification of nuclei has been achieved through density gradient fractionation using a sucrose gradient, a Percoll gradient, or a combination [48]. Yin and Komatsu [20] used a Plant Nuclei Isolation/Extraction kit (Sigma, St. Louis, MO, USA) with sucrose cushions in soybean subjected to submergence and succeeded to identify 365 proteins. A method that focuses on obtaining and analyzing pure nuclei has been published in barley [42]. The research group utilized flow cytometric sorting of cell nuclei to identify 803 nuclear proteins from cells at the G1 phase of the cell cycle (Table 1) [49]. Relatively pure fractions of nuclear compartment can be obtained as indicated by enzymatic activity measurement of other subcellular marker proteins, showing 2–7 times higher activities in total protein fraction [20]. Complex regulatory networks modulating gene expression await elucidation by detailed investigation of nuclear proteomics, especially in agronomically important non-model plants.

### 3.2. Mitochondria

Isolation of mitochondria in *Arabidopsis* employs differential centrifugation using sucrose gradients and Percoll gradients, which enables isolation of pure and intact organelles [50]. To further purify these organelles, the rice mitochondrial fraction is separated by free-flow electrophoresis [51]. Free-flow electrophoresis is carried out in an unsupported electrolyte flowing laminarly between two narrowly spaced glass plates, in which the analytes are streamed through a perpendicularly applied electric field. Using this purification method, differences between the mitochondrial proteome of root and photosynthetic shoots were recognized related to the tricarboxylic acid cycle and photorespiration [52]. Analysis of post-translational modification, namely Lys-Nε-acetylation, was also performed for the mitochondrial proteome of pea (Table 1) [53]. Enrichment of proteins that reside in the mitochondrial outer and inner membranes is necessary to dissect functional compartmentalization of individual membranes. In soybean, Komatsu et al. [25] utilized filtration through Miracloth (Calbiochem, San Diego, CA, USA) followed by mitochondrial purification by a QProteome Mitochondrial Isolation kit (Qiagen, Hilden, Germany). Purity check was done using markers for cytosol/mitochondria, mitochondria and chloroplast, respectively. Analysis of the mitochondrial proteome of soybean revealed a differential response between the outer and inner membranes (Table 1) [25]. Mitochondrion is a highly sensitive organelle that changes the coordination of the respiration chain in response to environmental stresses. Development and application of an efficient purification method for this organelle is essential to deepen our understanding of mitochondrial function.

### 3.3. Endoplasmic Reticulum

Purification of the endoplasmic reticulum is a complex and time-consuming process. Centrifugation through a discontinuous sucrose gradient is a method often utilized to isolate endoplasmic reticulum and Golgi apparatus from the cell lysate [63]. The endoplasmic reticulum and Golgi apparatus are then separated by free-flow electrophoresis [63]. To facilitate plant endoplasmic reticulum studies, the Endoplasmic Reticulum Enrichment kit (Novus, Littleton, CO, USA) which was developed primarily for animal materials, was adapted for soybean (Table 1) [11]. In this method, the cell lysate is centrifuged three times and 8 mM CaCl_2_ is added to the supernatant to precipitate endoplasmic reticulum membrane. After centrifugation, the pellet is collected as rough endoplasmic reticulum fraction [11]. Membranous cellular structures such as endoplasmic reticulum, Golgi apparatus and mitochondria are known to form a multifunctional membrane network [64]. Protein-protein interaction of reticulon-forming proteins on endoplasmic reticulum was analyzed by a proteomic technique and revealed how endoplasmic reticulum interacts with plasma membrane by affecting membrane curvature [65]. The simple method of Wang and Komatsu [11] allowed enrichment of ER proteins over 93% in each stress conditions with avoidance of contamination from other subcellular organelles which was assessed by enzymatic activity of marker proteins. This method can be a handy tool for further proteomic identification of unknown components involved in endoplasmic reticulum function.

### 3.4. Cell Wall

The cell wall is an important feature of plant cells that provides shape to different cell types [66]. The cell wall provides not only mechanical support to cells but also plays essential roles in plant adaptation to environmental cues, as reported for example during root elongation of sugarcane under drought stress (Table 1) [67]. Cell wall proteins representing about 5–10% of the cell wall mass are the main players behind this physiological adjustment. The most important feature in the purification of cell wall proteins is solubilization of proteins bound to the cell wall. Proteins that are loosely bound to the cell wall can be extracted with a series of solvents but efficient extraction of proteins that are tightly linked to the cell wall architecture can only be achieved using an appropriate extraction solution [68]. Solutions of 5 mM sodium acetate (pH 4.6) containing 200 mM CaCl_2_ or 2 M LiCl were used for cell wall protein extraction from sugarcane suspension cells (Table 1) [57] This method showed promising reproducibility in cell wall protein identification with 85% overlap among three replicates where 951 proteins identified at least in two replicates. For *Arabidopsis*, methods that can destroy cell walls have increased the number of identified cell wall proteins from 86 to 302, of which 27.5% act on polysaccharides in the stem; moreover, 361 proteins including 51 novel proteins were discovered in rosettes (Table 1) [58,59]. Examination of the structural and functional changes during development and stress responses in the cell wall is awaited.

### 3.5. Plasma Membrane

Plasma membrane intrinsic proteins are essential components of all signal transduction pathways; however, extraction and solubilization of these proteins for proteomic analysis are difficult because of their hydrophobicity, which allows them to reside in the plasma membrane lipid bilayer [69]. Two-phase partitioning utilizing the different surface properties of membranes was applied to isolate membrane proteins and highly hydrophobic proteins including V-ATPase subunits and transmembrane proteins in *Arabidopsis* [69]. Mitra et al. [70] introduced chloroform extraction as a step after phase partitioning and obtained a 2-fold increase in detection of membrane transporters. Phase partitioning was combined with free-flow electrophoresis, leading to identification of 1029 proteins from *Arabidopsis* seedlings (Table 1) [60]. The purity of isolated plasma membrane proteins was high enough to bring about the discovery of thirteen peripheral and soluble proteins associated with the plasma membrane [60]. Moreover, plasma membrane proteins were identified that take part in signal transduction triggering innate immunity using a stress-induced change in membrane properties [71]. Detergent-resistant membranes are specialized microdomains in plasma membranes characterized by their insolubility in non-ionic detergents at 4 °C [72]. Purification and analysis of rice proteins from this region indicated that detergent-resistant membranes function as a place for multiprotein signaling complex formation, as indicated by the detection of OsRac1 and RACK1A during activation of chitin-mediated innate immunity [71]. Applying a suitable purification method at an appropriate phase of biological phenomena will deliver more information on the dynamics of biological membranes and the delicate mechanisms underlying plant stress responses

### 3.6. Validation of Purity

The isolation and purification of cellular organelles allows the identification of compartment specific proteins. However, the accuracy of such identification is dependent on the purity of the targeted organelle and is also influenced by the degree of protein enrichment and extent of contamination. A variety of methods have been developed and widely applied for purity assessment including both microscopic and biochemical methods. To perform these methods, a sufficient level of expertise is required and only a few dyes specific for subcellular organelles are commercially available. As an alternative approach for purity assessment, enzymatic and Western blot analyses have been developed.

For Western blot analysis, specific antibodies against subcellular marker proteins are needed. For example, histone 3, mitochondrial ascorbate peroxidase and calnexin were used to estimate the purity of nuclear, mitochondrial and endoplasmic reticulum fractions, respectively. In addition, cytosolic ascorbate peroxidase was used to estimate contamination of the cytosol [24,26]. However, compared to Western blot analysis, enzyme assays have proved to be a more convenient approach to evaluate subcellular organelle purity. A number of articles have described the known enzymes that are suitable for assays of subcellular compartment purity.

The purity of the plasma membrane was estimated by comparing H^+^-ATPase activity to the total ATPase activity [28,72]. Assay of histone acetyltransferase activity, which was monitored at 340 nm, was used to estimate the purity of the nuclear fraction [73]. The purity of mitochondria has been estimated using a number of enzymes, including fumarase, aconitase and cytochrome *c* oxidase [74,75,76]. For evaluating the purity of chloroplasts, assays for phosphoribulokinase activity [75] and NADH-dependent glyceraldehyde 3-phosphate dehydrogenase activity have been reported [74,77]. For the endoplasmic reticulum, NADH: cytochrome *c* reductase activity is commonly used to assess purity [78,79]. Furthermore, the activity of glucose-6-phosphate dehydrogenase [80] can be used to estimate cytosolic contamination. Because light and electron microscopy, Western blotting and enzyme analyses have comparative advantages and disadvantages, it is ideal to use multiple methods for purity assessment.

## 4. Subcellular Proteomics in Soybean under Flooding Stress

### 4.1. Nuclear Proteomics in Soybean under Flooding Stress

To identify the upstream events controlling the regulation of flooding-responsive proteins, a nuclear proteomics was performed. Komatsu et al. [21,81] and Oh et al. [23] respectively reported identifying 65, 39 and 95 nuclear proteins without the use of Percoll or sucrose cushions in response to flooding stress. Yin and Komatsu [20] identified 365 nuclear proteins using the Plant Nuclei Isolation/Extraction kit with sucrose cushions; of these, these proteins overlapped with 13 of the 65, 7 of the 39 and 27 of the 95 proteins identified in the earlier studies, which only used immunoblotting to confirm the purity of the enriched nuclei fraction. On the other hand, immunoblotting and enzyme activity analyses clearly indicated that the nuclear proteins were highly enriched and had low contamination from other subcellular proteins [20]. These results indicated that the purity of enriched soybean nuclei is improved by using Percoll and sucrose cushions and that enzyme assays of subcellular markers are a more useful approach to confirm purity compared with only immunoblotting analysis.

At the initiation of flooding stress, protein translation is suppressed through decreased abundance of proteins related to preribosome biogenesis and mRNA processing in the nucleus of soybean root tip. Furthermore, 17 chromatin structure-related nuclear proteins were decreased in response to flooding; especially, histone H3 was clearly decreased with protein abundance and mRNA expression levels. These results indicated that flooding stress may regulate histone variants through gene expression in root tip [20]. For further analysis, phosphopeptides in the fractions were enriched using Polymer-based Metal-ion Affinity Capture Phosphopeptide Enrichment Reagent (Tymora, West Lafayette, IN, USA) and a phosphoproteomic analysis was performed [16]. It is indicated that protein synthesis-related proteins in the nucleus that respond to abscisic acid were identified as phosphoproteins at the initial stage of flooding stress [16]. When abscisic acid is added to soybeans during flooding, survival is improved [81], suggesting that abscisic acid might be involved in the enhancement of flooding tolerance. These findings suggest that transcription, preribosome biogenesis and mRNA processing related proteins are suppressed, despite the increased phosphorylation of protein synthesis-related proteins at the initiation of flooding.

### 4.2. Mitochondrial Proteomics in Soybean under Flooding Stress

Flooding decreases the oxygen concentration in plant surroundings, which in turn restricts ATP production via mitochondrial oxidative phosphorylation [82]. To understand mitochondrial function, mitochondrial proteomics has been conducted. Komatsu et al. [25] used two-dimensional polyacrylamide gel electrophoresis and blue native polyacrylamide gel electrophoresis and the protein spots regulated in response to flooding stress were identified using MS. On the other hand, Kamal and Komatsu [24] and Mustafa and Komatsu [83] used a gel-free/label-free proteomic technique. Komatsu et al. [25] reported detecting 72 mitochondrial membrane protein spots and 327 matrix protein spots. Kamal and Komatsu [24] and Mustafa and Komatsu [83] identified 98 mitochondrial proteins using Percoll gradient methods and 713 using the QProteome Mitochondrial Isolation kit. For isolation of plant mitochondrial fractions, the QProteome Mitochondrial Isolation kit is convenient because there is no necessity for an ultracentrifuge.

Using a mitochondrial proteomic technique, it was confirmed that flooding stress directly impairs electron transport chains, though NADH production increases in the mitochondria through the tricarboxylic acid cycle in root including hypocotyl [25]. Kamal and Komatsu [24] analyzed mitochondrial proteins in soybean root under flooding stress to understand the mechanism of biophoton emission; their results suggested that oxidation and peroxide scavenging lead to biophoton emission and oxidative damage under flooding stress. Mustafa and Komatsu [83] performed mitochondrial proteomics, because the mitochondrion was the target organelle of aluminum oxide nanoparticles under flooding stress. Their results indicated that aluminum oxide nanoparticles of various sizes affect mitochondrial proteins by regulating membrane permeability and tricarboxylic acid cycle activity under flooding stress. These findings provide insight into the effect of flooding on mitochondrial function in early-stage soybean and reveal that several mitochondrial proteins may play a role in the mitochondrial response to flooding stress.

### 4.3. Endoplasmic Reticulum Proteomics in Soybean under Flooding Stress

Flooding stress has a severe effect on endoplasmic reticulum function due to changes in the levels of calnexin, heat shock protein 70 and luminal binding protein [13]. Furthermore, flooding stress might negatively affect *n*-glycosylation of proteins related to stress and protein degradation; however, glycoproteins involved in glycolysis are activated [10]. Based on this knowledge, proteins in endoplasmic reticulum were analyzed. Using the Endoplasmic Reticulum Enrichment kit and a gel-free proteomic technique, Komatsu et al. [26] identified 117 and 212 proteins with increased and decreased abundance, respectively and Wang and Komatsu [11] identified 368 changed proteins. Furthermore, 74 ribosomal proteins were identified because these studies used purification methods for rough endoplasmic reticulum.

Using an endoplasmic reticulum proteomic analysis, Komatsu et al. [26] suggested that flooding stress mainly affects protein synthesis and glycosylation in the endoplasmic reticulum in soybean root. Furthermore, Wang and Komatsu [11] compared endoplasmic reticulum function after flooding and drought stresses. They suggested that reduced accumulation of glycoproteins in response to both stresses might be due to dysfunction of protein folding through the calnexin/calreticulin cycle. Additionally, the increased cytosolic calcium level induced by both stresses might disturb the endoplasmic reticulum environment for proper protein folding in soybean root tip [11]. Taken together, these results indicate that the endoplasmic reticulum is significantly affected in soybean under flooding stress, with protein glycosylation, protein degradation and calcium release.

### 4.4. Cell Wall and Plasma Membrane Proteomics in Soybean under Flooding Stress

The primary cell wall plays a role in regulating extension growth, cell adhesion and cell morphology [84]. Pereira et al. [85] reported that the primary cell wall is essential for a hydrated matrix and responds sensitively to global changes in the water content of the plant by altering its biophysical properties. Partial adaptation to flooding stress might require a compensatory modification of the extracellular matrix [22]. To investigate the function of the soybean cell wall under flooding stress, cell wall proteomics was performed [27]. Based on a gel-based proteomic technique, 16 out of 204 cell wall proteins responded to flooding stress, which included two lipoxygenases, four germin-like proteins, three stem glycoproteins and a Cu-Zn superoxide dismutase [27]. These changed proteins indicated that flooding stress suppresses lignification through decreasing the levels of these cell wall proteins by downregulation of reactive oxygen species and inhibition of jasmonate biosynthesis in the root.

The plasma membrane is considered an important player in anoxia stress during flooding. Cytosolic pH regulates root water transport during anoxic stress through gating of aquaporins, which are water channel proteins intrinsic to the plasma membrane [86]. Using gel-based proteomic technique, although only superoxidase dismutase was identified, proteins related to the antioxidative system in plasma membrane and cytosol might play a crucial role in protecting cells from oxidative damage. Additionally, heat shock cognate protein plays a role in protecting proteins from denaturation during flooding stress; and signaling related proteins might regulate ion homeostasis [28]. Both of these proteomic analyses of cell wall and plasma membrane were performed using gel-based proteomics; however, if a gel-free proteomic technique is used, more important roles of cell wall and plasma membrane in flooding responses may be clarified.

## 5. Construction of a Subcellular Proteomics Related Database

To provide information on proteins for functional analyses, the Soybean Proteome Database was originally constructed using data for soybean proteins separated by two-dimensional polyacrylamide gel electrophoresis, a gel-based proteomic technique [46] (http://proteome.dc.affrc.go.jp/Soybean/). In addition, data were integrated using information from transcriptomics, proteomics and metabolomics [87]. The database has been improved by incorporation of data from label-free MS-based quantitative proteomics, a gel-free proteomic technique [88] and is linked to a soybean genome database, DAIZUbase [89] (http://daizu.dna.affrc.go.jp/). The Soybean Proteome Database also stores information on flooding-responsive proteins, which were analyzed in temporal and organ-specific protein profiles of soybean grown in the presence or absence of flooding stress [88]. Furthermore, these data can be searched from PlantPReS, a database for plant proteome responses to stress, which includes information such as protein name, accession number, plant type, tissue, organelle, stress type and developmental stage [90] (www.proteome.ir). These data should be useful for analyses of biological mechanisms utilized in soybean, especially coupled with recent advances in information and communication technology.

## 6. Conclusions

**Figure 1:**
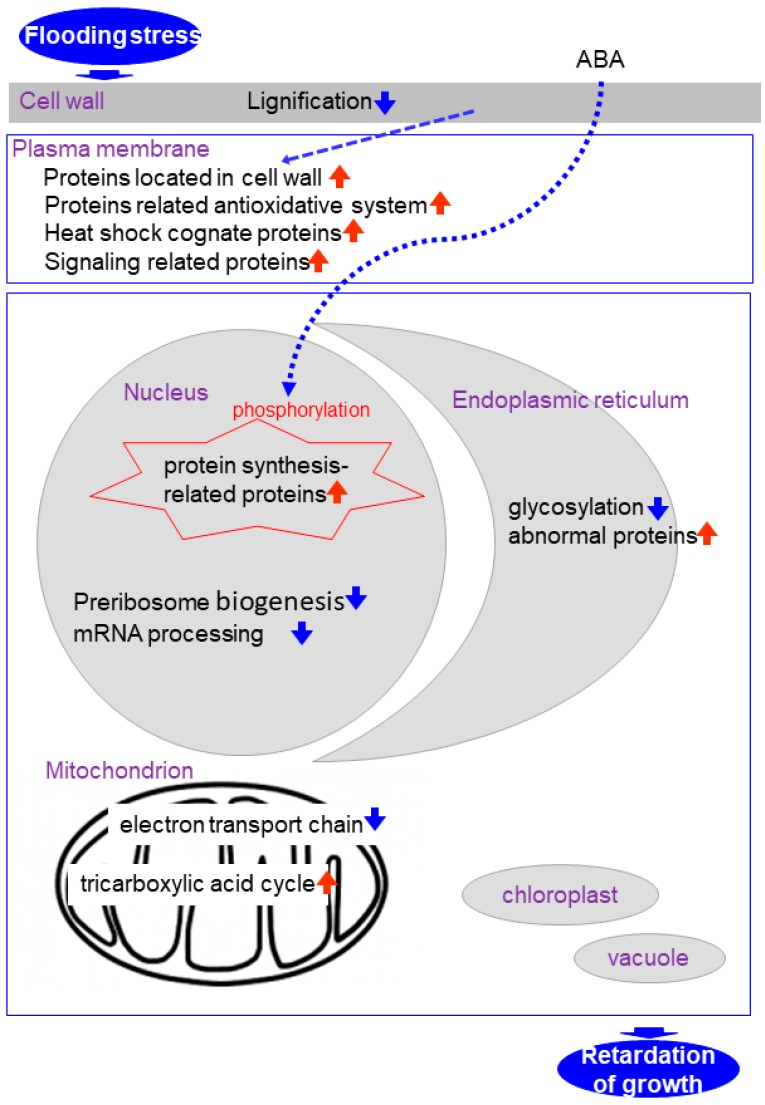
Representation of the cross talk among different pathways in soybean under flooding condition.

Proteomics is a reasonable tool for the elucidation of flooding response mechanisms in soybean. Subcellular proteomics has the potential to elucidate localized cellular responses and investigate communications among subcellular components during plant growth and under stresses. Under flooding, proteins related to signaling, stress and the antioxidative system are increased in the plasma membrane; reactive-oxygen species scavenging enzymes are suppressed in the cell wall; protein translation is suppressed through inhibition of preribosome biogenesis- and mRNA processing-related proteins in the nucleus; proteins involved in the electron transport chain are reduced in the mitochondrion; and protein-folding related proteins are decreased in the endoplasmic reticulum. In the case of flooding response in early-stage soybean the subcellular compartments described above were examined because they are significantly damaged (Figure 1). However, because the chloroplast and vacuole are also important organelles for plants, proteomic analyses of them are also necessary. This work will expedite transgenic or marker-assisted genetic enhancement studies in crops for developing high-yielding stress-tolerant lines or varieties under abiotic stress.

## Figures and Tables

**Table 1:** Techniques used for cellular organelles isolation in plant subcellular proteomic studies.

Organ	Species	Purification	Proteome Analysis	Identified Proteins	Representative Ref
*Nucleus*					
Cell culture	*Arabidopsis*	Density gradient	LC-MS/MS	2544 proteins	[54]
Aerial parts	Chickpea	Density gradient	2DE LC-ESI-MS/MS, MALDI-TOF/TOF	107 phosphoproteins	[52]
Seedlings	*Pinus radiata*	Density gradient	LTQ-Orbitrap MS	33 transcription factors/regulators	[55]
Grains	Barley	Flow cytometric sorting	1DE LC-MS/MS MALDI-MS/MS	803 nuclear proteins	[49]
*Mitochondrion*					
Shoot	Rice	Density gradient, Free-flow electrophoresis	Gel based/LC-MS/MS	322 proteins	[56]
Seedlings	Pea	Density gradient	LC–MS/MS	358 Lys-Nε-acetylated proteins	[53]
Root/hypocotyl	Soybean	QProteome Mitochondrial Isolation kit	2DE, LC–MS/MS	327 proteins	[25]
*Endoplasmic reticulum*					
Root tip	Soybean	Endoplasmic Reticulum Enrichment kit	LC–MS/MS	255, 368, 103 proteins in control, flooding, drought	[11]
*Cell wall*					
Cell culture	Sugarcane	Washings of cell walls with 5 mM acetate buffer	1DE, LC–MS/MS	377 proteins	[57]
Mature stem	*Arabidopsis*	Washings of cell walls with 5 mM acetate buffer	1DE, LC–MS/MS	302 cell wall proteins	[58]
Rosettes	*Arabidopsis*	Washings of cell walls with 5 mM acetate buffer	1DE, LC–MS/MS	361 cell wall proteins	[59]
*Plasma membrane*					
Seedlings	*Arabidopsis*	Density gradient, Free-flow electrophoresis	LC–MS/MS	1029 proteins	[60]
Seedlings	*Arabidopsis*	Density gradient	2D-DIGE, LC-MS/MS, MALDI-TOF/MS	36 microdomain proteins	[61]
Seedlings	Oat, Rye	Density gradient	LC–MS/MS	740, 809 proteins in oat, rye	[62]

## Abbreviations

LC	liquid chromatography
MS	mass spectrometry
UPR	unfolded protein response
